# *Leptomischus hiepii*, a new species of Rubiaceae from Vietnam

**DOI:** 10.3897/phytokeys.166.55731

**Published:** 2020-11-13

**Authors:** Lei Wu, Leonid V. Averyanov, Khang Sinh Nguyen, Tatiana V. Maisak, Yan-Hua Hu

**Affiliations:** 1 College of Forestry, Central South University of Forestry and Technology, Changsha 410004, China Central South University of Forestry and Technology Changsha China; 2 Komarov Botanical Inst., Russian Academy of Sciences, Prof. Popov St. 2, RU-197376 St. Petersburg, Russia Komarov Botanical Inst., Russian Academy of Sciences St. Petersburg Russia; 3 Institute of Ecology and Biological Resources, Vietnam Academy of Sciences and Technology, 18 Hoang Quoc Viet, Cau Giay, Ha Noi, 100000, Vietnam Institute of Ecology and Biological Resources, Vietnam Academy of Sciences and Technology Ha Noi Vietnam; 4 Graduate Univ. of Science and Technology, Vietnam Academy of Science and Technology, Hanoi, 100000, Vietnam Graduate Univ. of Science and Technology Ha Noi Vietnam

**Keywords:** *
Argostemmateae
*, endemism, plant diversity, plant taxonomy

## Abstract

*Leptomischus hiepii*, a new species of the tribe Argostemmateae from Son La province, northwestern Vietnam, is described and illustrated. Morphologically it allies to *L. wallichii*, *L. erianthus* and *L. funingensis* by sharing a similar habit, large stipules and similar corolla shape, but it differs by its anisophyllous leaves, 1-flowered inflorescences, homostylous flowers and tubular-campanulate corollas.

## Introduction

*Leptomischus* Drake (Drake 1895) is a poorly known genus of Rubiaceae. It was first described as a monotypic genus with one species, *L. primuloides* Drake (Drake 1895), occurring in northern Vietnam. Nearly a century later, Lo (1986) published the second species found in southern and southwestern China and northern Vietnam, i.e. *L. parviflorus* H.S.Lo. Later, in his revision of *Leptomischus*, Lo (1993) included *Indopolysolenia* Bennet (Raizada and Bennet 1981) as a synonym of *Leptomischus* and accommodated a new combination, *L. wallichii* (Hook.f) H.S.Lo for *Polysolenia wallichii* Hook.f. (Bentham and Hooker 1873) from India. He also proposed a new synonym of *L. primuloides*, i.e. *Indopolysolenia burmanica* Deb & Rout (Deb and Rout 1990) reported from northern Myanmar. Subsequently, Lo (1998) reported three more new species of *Leptomischus* endemic to southwestern China; *L. erianthus* H.S.Lo, *L. funingensis* H.S.Lo, and *L. guangxiensis* H.S.Lo. Recently, *L. flaviflorus* Hareesh, L.Wu & M.Sabu, a new species from southern Tibet and northeastern India was published (Hareesh et al. 2017). To summarize, *Leptomischus* presently includes seven species distributed in tropical mainland Asia, including northeastern India (2 species), Myanmar (1 species), southern and southwestern China (5 species), and northern Vietnam (2 species).

Despite the fact that the genus *Leptomischus* is insufficiently known and has previously been studied by few researchers (Lo 1986, 1993, 1998; Chen and Taylor 2011; Hareesh et al. 2017), this group of plants can be readily distinguished from other genera of Rubiaceae by a combination of the following characters: perennial herbs or shrubs, heterostylous flowers, numerous ovules which are borne on a stipitate placenta near the base of the septum, dry capsular fruits dehiscing through the apical portion or through an operculum, small and numerous seeds with reticulate testa (Lo 1993; Chen and Taylor 2011; Razafimandimbison and Rydin 2019). *Leptomischus* is considered to belong to the Asian tribe Argostemmateae in subfamily Rubioideae and was found to be sister to all other genera of the tribe (Razafimandimbison and Rydin 2019).

Among the characteristic features mentioned above, the mode of dehiscence of the capsule is the most important, as only two genera of the tribe share such a character (Lo 1993), i.e. *Leptomischus* and *Mouretia* Pitard (Pitard 1922). It is said that placenta insertion (near the base of the septum in *Leptomischus* and near the middle of the septum in *Mouretia*) is a key character to distinguish *Leptomischus* from *Mouretia* (Chen and Taylor 2011; Hareesh et al. 2017). However, the insertion of the placenta is difficult to observe since the ovary is quite small and the placentas are large in comparison with the septum. As a result, this characteristic was not illustrated for any species described (Bentham and Hooker 1873; Drake 1895; Raizada and Bennet 1981; Deb and Rout 1990; Lo 1986, 1993, 1998; Chen and Taylor 2011; Hareesh et al. 2017). Moreover, while describing *Leptomischus* in China, Chen and Taylor (2011) stated that the placentas were “inserted apparently near the middle of septum”, and we also found that the placenta of *L. funingensis* is inserted at the middle of septum (Fig. 4I). To our knowledge, all representatives of *Leptomischus* have corolla tubes longer than 14 mm (except for 6–6.5 mm long in *L. parviflora*), anthers 2–3.5 mm long and stigmas 2–5 mm long (Deb and Rout 1990; Lo 1998; Chen and Taylor 2011; Barbhuiya et al. 2014; Hareesh et al. 2017), whereas species of *Mouretia* have corolla tubes shorter than 4 mm, anthers 0.9–1.4 mm long and stigmas 0.7–2 mm long (Tange 1997; Chen and Taylor 2011).

During an expedition to Muong La Nature Reserve in Son La Province of northern Vietnam, an unusual species of the family Rubiaceae was discovered. It is a perennial herb, mostly glabrous, with anisophyllous leaves, usually 2-lobed stipules, terminal 1-flowered inflorescences, actinomorphic, 5–6-merous, hermaphroditic homostylous flowers, numerous ovules on a stipitate placenta attached to the middle of the septum, fleshy capsular fruits dehiscing through apical portion, small and numerous seeds with reticulate or verrucose testa. It clearly belongs to the tribe Argostemmateae, in which all genera are characterized by hermaphroditic flowers, bilocular ovaries (sometimes 3–5-celled in *Mycetia* Reinw., capsular fruits, and many small seeds (Rydin et al. 2009; Ginter et al. 2015; Razafimandimbison and Rydin 2019). Because of the corolla tube (3.2–)3.4–3.8(–4) cm long and (1.2–)1.3–1.5(–1.7) cm in diameter at the throat, the anthers 2.2–2.5 mm long, the stigmas 2.5–3 mm in length and the capsular fruits dehiscing through the apical portion, we tentatively place it in the genus *Leptomischus*.

After consulting the relevant literature (Bentham and Hooker 1873; Deb and Rout 1990; Lo 1986, 1993, 1998; Pham 2003; Barbhuiya et al. 2014; Chen and Taylor 2011; Hareesh et al. 2017) and available herbarium specimens (including types) housed in Vietnam (HN, VNMN) and China (BNU, CSFI, GXMI, HITBC, IBK, IBSC, KUN, PE), and digital images of specimens at K (https://apps.kew.org/herbcat/navigator.do) and P (https://science.mnhn.fr/all/search), the discovered plants were found to represent an undescribed species. The description and illustration of this new species is provided below.

## Material and methods

The specimens of the newly described species are deposited at the following herbaria: Central South University of Forestry and Technology (CSFI), Institute of Ecology and Biological Resource of the Vietnam Academy of Science and Technology (HN), and Komarov Botanical Institute of Russia (LE). Herbarium acronyms follow Thiers (2020). Morphological observations and measurements of the new species are based on living material in the field as well as on dry specimens. The conservation status of the new species is evaluated based on field observations in accordance with IUCN guidelines (2017).

## Taxonomic treatment

### Leptomischus hiepii

Taxon classificationPlantaeGentianalesRubiaceae

L.Wu, K.S. Nguyen & Aver. sp. nov.

1F04817C-65EE-521B-BE01-9216B13A2C07

urn:lsid:ipni.org:names:77212876-1

**Figure 1.**
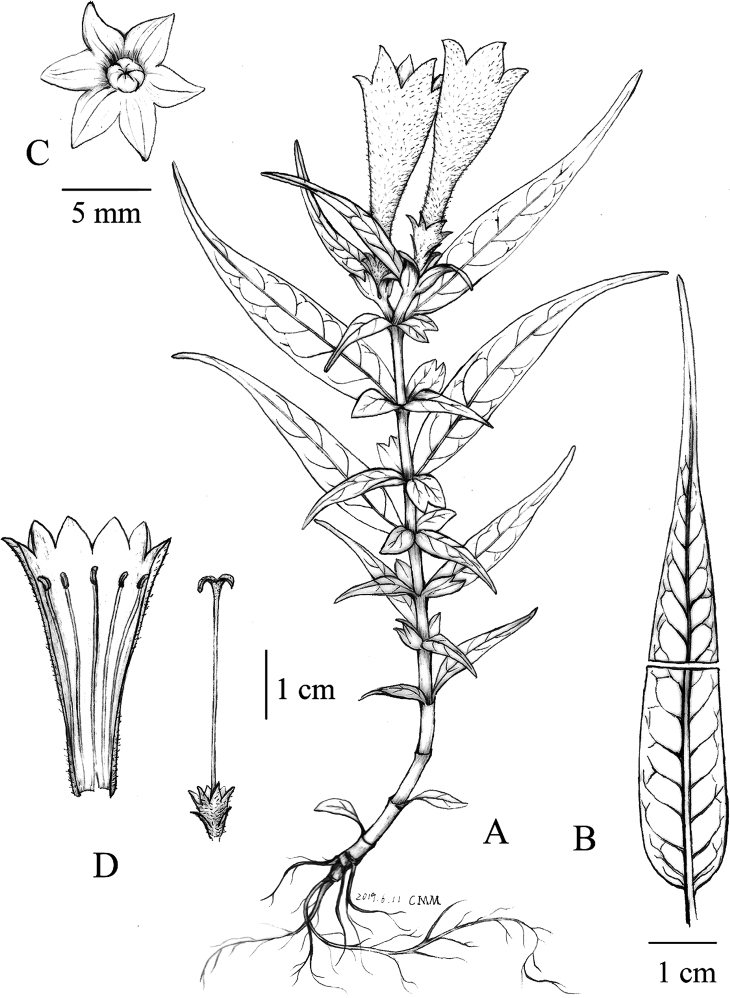
*Leptomischus hiepii***A** habit **B** leaf, upper portion, adaxial surface; lower portion, abaxial surface **C** capsule, view from above **D** dissected flower showing stamens and pistil. Drawn by Bi-Shan Li from the holotype in CFSI.

**Figure 2.**
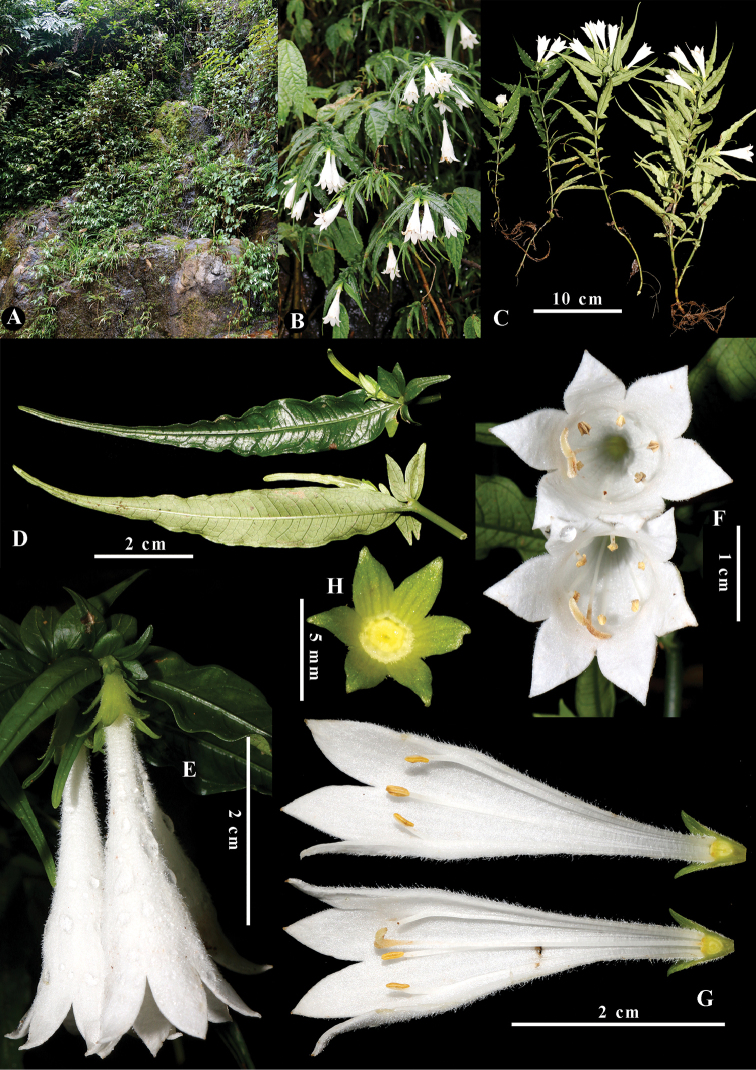
*Leptomischus hiepii***A** typical habitat **B, C** habit **D** unequal leaf pair and stipules **E** inflorescence **F** flowers, view of the throat **G** dissected flower **H** calyx lobes and disc, seen from above. Photos and design by K.S. Nguyen and L. Wu.

**Figure 3.**
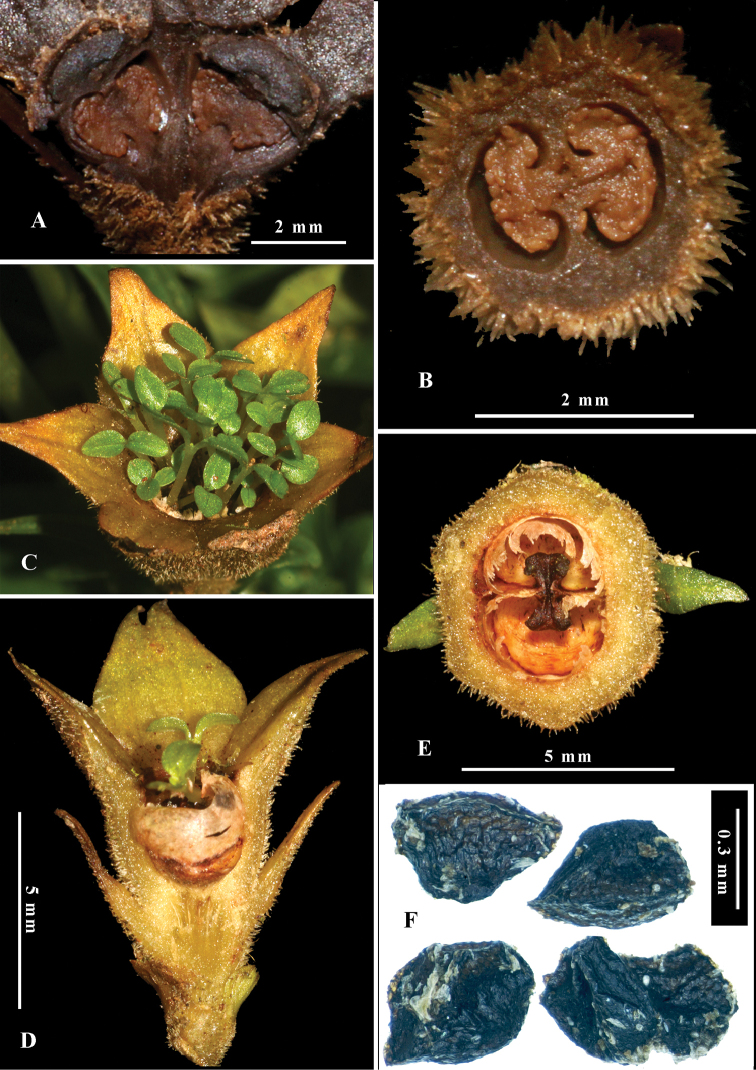
*Leptomischus hiepii***A** longitudinally dissected ovary **B** transversely dissected ovary **C** mature fruits and viviparous seeds **D** longitudinally dissected capsule **E** Transversely dissected capsule **F** seeds. Photos and design by K.S. Nguyen.

Figs 1 , 2 , 3 , Table 1

#### Diagnosis.

Similar to *L. wallichii*, *L. erianthus* and *L. funingensis*, but differs mainly by its anisophyllous leaves (vs. isophyllous), stipules usually 2-lobed (vs. entire or 3-lobed), 1-flowered inflorescence (vs. several-flowered), homostylous flowers (vs. heterostylous), and corolla tubular-campanulate, 3.2–4 cm long (vs. tubular, tubular-infundibulariform or tubular-salverform, 1.4–1.6(–3) cm).

#### Type.

Vietnam. Son La province: Muong La district, Ngoc Chien commune, Muong La Nature Reserve, around point 21.61032°N, 104.10576°E, elevation 1320–1350 m a.s.l., remnants of primary evergreen broad-leaved forest along streams at base and on steep slope of sandstone mountain, lithophytic perennial herb 30–45 cm tall, in wet places, locally common, 2 March 2019, *Nguyen Sinh Khang, Mai Van Duc and Lo Van Chieu, NSK 1153* (holotype CSFI [CSFI069614]; isotypes CSFI, HN, LE [LE01058686, LE01058688, photo: LE01061374]).

#### Description.

Perennial herb, lithophytic and occasionally terrestrial, 30–45 cm tall. Stem ascending or drooping, somewhat straight, simple or branched, terete, glabrous, green, with internodes of 6–30 mm long, 2–3 mm in diameter. Stipules persistent, glabrous, green, slightly equal to unequal, broadly to narrowly ovate, (4–)8–10(–16) × (3–)4–8(–10) mm, distally often divided up to 2/3^th^ of their length into 2 narrowly ovate to broadly lanceolate, 3–5(–9) mm long obtuse lobes, usually bearing 2–3 longitudinal veins united at base and 3–5 lateral veins on each side. Leaves anisophyllous, glabrous on both surfaces, dark green above, paler green below, subsessile to shortly petiolate; petioles terete, 0.5–2.5 mm long, glabrous; leaf blades lanceolate to narrowly lanceolate, cuneate at base, gradually attenuate to caudate at apex, margin entire, often irregularly wavy; veins prominent on both surfaces; of a pair of leaves, the smaller one (8–)10–20(–30) × (2.5–)3–5(–8) mm, with (3–)5–7(–9) secondary veins on each side of midrib, the larger one (3–)5–9(–11) × (0.4–)0.8–1.2(–1.5) cm, with (9–)11–15(–17) secondary veins on each side of midrib. Inflorescence terminal, single-flowered; peduncle ca. 1 mm long, densely puberulent; bracts 2, subulate, 5–6 mm long, ca. 1 mm wide at base, acute, puberulent outside, glabrous inside. Flowers solitary, sessile, 6-merous, sometimes 5-merous, bisexual, homostylous. Calyx campanulate, densely puberulent outside; hypanthium obconic, 2.5–3.2 mm long, lobes triangular, acute, 2.2–4 × 1.6–2.5 mm, almost glabrous inside, ciliate along margin, somewhat recurved at anthesis. Corolla tubular-campanulate, (3.2–)3.4–3.8(–4) cm long, (1.2–)1.3–1.5(–1.7) cm in diameter at the throat, pure white, shortly densely villous outside, almost glabrous inside; corolla lobes (5–)6, triangular ovate, straight spreading or slightly recurved at anthesis, 5–8 mm × 4–7 mm, tips acute. Stamens (5–)6, filaments white, glabrous, connate with corolla tube from base to 5–6 mm below the throat, free parts terete, 1.8–2.6 mm, slightly incurved; anthers pale yellowish, oblong elliptic, 2.2–2.5 × 0.6–0.8 mm, dorsifixed, introrse. Ovary inferior, bilocular; disk glabrous, marginally convex and concave at the center; style erect, filiform, 2.5–3.2 cm long, white, glabrous; stigma dull brownish, finely papillose, 2-lobed; lobes narrowly oblong or linear, 2.5–3.5 mm long, recurved at a straight angle from the style axis, positioned 1.4–1.8 mm below the throat, slightly above anther apices. Fruit capsular, subglobose, ca. 3–4 mm in diameter, crowned by persistent calyx lobes, dehiscent through apical portion, pericarp and septum membranous, brown; placenta fleshy, brown, mushroom-shaped during anthesis then turning dark brown, woody, and broadly conoid (when dried), attached to middle of septum, distally bearing numerous seeds; seeds angled, 0.3–0.5 mm; testa reticulate or verrucous, black.

**Table 1.** Morphological comparison of *Leptomischus hiepii*, *L. wallichii*, *L. erianthus* and *L. funingensis* (Lo 1993; Chen and Taylor 2011; Hareesh et al. 2017).

Characters	*L. hiepii*	*L. wallichii*	*L. erianthus*	*L. funingensis*
Stem	glabrous	Glabrous	densely hirsute	Pilose
Stipule	narrowly ovate to broadly ovate, 8–14 mm long, entire to 2-lobed	ovate to lanceolate, 7–13 mm long, 3-lobed	ovate or lanceolate, 8–9 mm long, entire	suborbicular, 4–5 mm long, entire
Leaves	anisophyllous, lanceolate to narrowly lanceolate, glabrous, 0.8–11 × 0.25–1.5 cm	isophyllous, oblanceolate to elliptic, glabrous, 4–12 × 0.7–1.8 cm	isophyllous, narrowly elliptic to lanceolate-elliptic, strigose to glabrescent, 4–12 × 1.5–4 cm	isophyllous, ovate, narrowly elliptic, or rarely obovate, adaxially glabrescent to pilose, abaxially villose, 8–15 × 2.5–4.5 cm
Inflorescence	1-flowered	capitate, several-flowered	subcapitate, several-flowered	cymose, several-flowered
Flower	homostylous, (5–)6-merous	unknown, 5-merous	distylous, (4–)5(–7)-merous	distylous, (4–)5(–6)-merous
Corolla shape	tubular-campanulate	Tubular	tubular-infundibulariform	tubular-salverform
Corolla tube length	3.2–4 cm	ca.1.5–3.0 cm	1.5–1.6 cm	1.4–1.6 cm
Anther length	2.2–2.5 mm	ca. 2 mm	2–2.5 mm	2–2.2 mm
Stigma length	2.5–3.5 mm	ca. 1.3–1.5 mm	3–3.5 mm	3.5–4.5 mm

#### Etymology.

The specific epithet honors Dr. Nguyen Tien Hiep, a famous botanist who made significant contributions to the plant taxonomy and nature conservation in Vietnam.

#### Additional specimens examined

**(paratypes)**. Vietnam. Son La province: Muong La district, Ngoc Chien commune, Muong La Nature Reserve, same location as type specimen, lithophytic perennial herbs bearing fleshy capsules, fruit opened by an apical operculum, 22 August 2020, *Nguyen Sinh Khang & Lo Van Chieu, NSK 1347* (HN).

#### Habitat, phenology and conservation status.

Lithophytic or terrestrial herb growing on rocks in streams and on moist cliffs under primary and secondary evergreen broad-leaved submontane forest on sandstone at elevations of 1300–1400 m a.s.l.. The plants flower in February–March, and bear fruits in July–August. The species was observed as being very common on waterfall cliffs and in humid places; around 300–500 mature individuals occur in Muong La Nature Reserve, spread over a very limited area of approximately 2500 m^2^. In Muong La Nature Reserve, agricultural activities and exploitation for timber and non-timber forest products are prohibited. According to indigenous people, this species is not used as medicinal or ornamental plant, and disturbances to its existence so far have not been recorded. Numerous localities in the mountainous areas of the Hoang Lien Son range, spreading over the provinces of Lai Chau (Phong Tho, Tam Duong and Than Uyen districts) and Lao Cai (Bat Xat, Sa Pa and Van Ban districts) to Yen Bai (Tram Tau, Nghia Lo, Van Yen and Mu Cang Chai districts) and Son La (Muong La and Bac Yen districts), in north-western Vietnam and the south-western parts of Yunnan province in south-western China, fit the habitat characteristics of this new species. It is therefore expected that more populations of the species will be discovered soon if extensive field surveys are carried out in this region. At present, however, *L. hiepii* can be considered as an endemic species to Son La province, and is tentatively assessed as “data deficient” (DD) in accordance with the IUCN Red list categories and criteria (2017).

#### Distribution.

Endemic to north-western Vietnam (Son La province, Muong La district, Muong La Nature Reserve).

#### Discussion.

**Figure 4.**
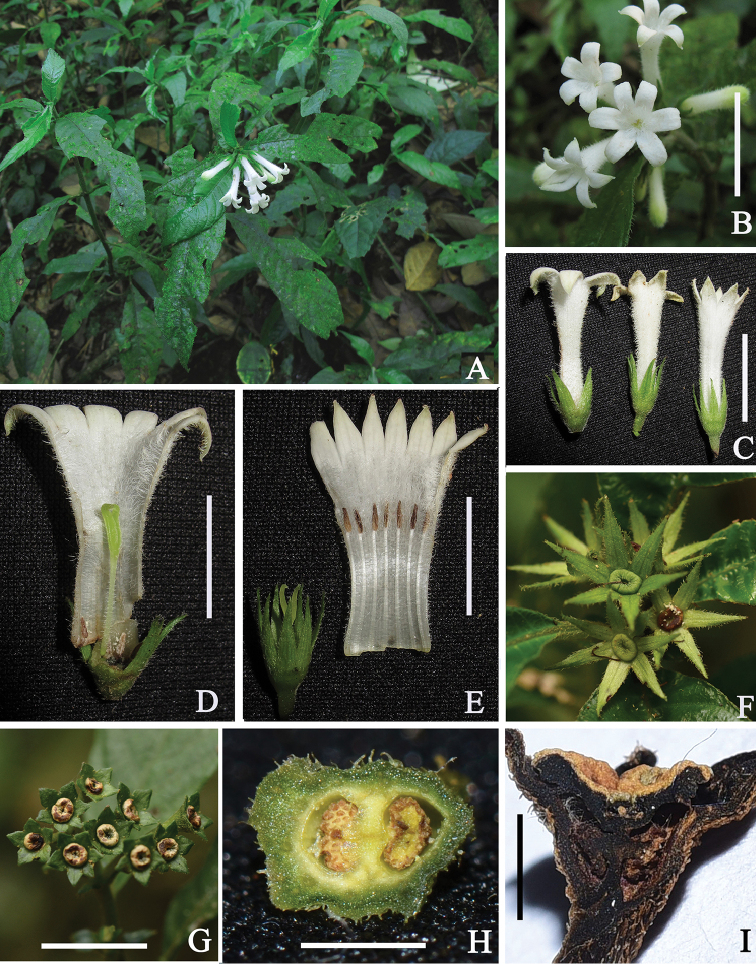
*Leptomischus erianthus***A** habit **B** inflorescence **C** flowers **D** Dissected longistylous flower **E** dissected brevistylous flower **F** capsules, seen from above. *L. funingensis*. **G** infructescence, seen from above **H** transversely dissected ovary **I** Longitudinally dissected ovary. Scale bars: 1 cm (**A–G**); 2 mm (**H, I**). Photos and design by L. Wu.

*Leptomischus hiepii* is a very special member of the genus in several ways, and is inconsistent with most of its congeners. Typical characters of *L. hiepii* are anisophyllous leaves, 1-flowered inflorescences, homostylous (5–)6-merous flowers and anthers and stigma positioned at the level of the throat of the corolla tube, while all currently known species of the genus are reported as having isophyllous leaves, several-flowered inflorescences, heterostylous 5-merous flowers, and anthers positioned much lower than the stigma or vice versa. Anisophyllous and isophyllous leaves are commonly seen in *Mouretia*, *Mycetia* and *Argostemma* Wall. within the tribe Argostemmateae (Chen and Taylor 2011). The 1-flowered inflorescence has not been recorded in *Leptomischus* but is reported in *Argostemma*, e.g. in *A. bachmaense* T.V.Do (Do et al. 2020). Homostylous flowers have hitherto not been recorded in the tribe Argostemmateae but the rare presence of homostylous flowers in otherwise heterostylous genera is not rare in Rubiaceae (Chen and Taylor 2011), e.g., homostylous flowers are reported in *Mussaenda campanulata* T.T.Duan & D.X.Zhang (Duan et al. 2016) and *Ophiorrhiza longifloriformis* Schanzer (Schanzer 2005). Flower merosity is variable in the genera *Mycetia*, *Argostemma* (Chen and Taylor 2011) and *Leptomischus* as well, or even within the same inflorescence of a species such as *L. erianthus* bearing 4–7-merous flowers (Fig. 4B, E). Anthers and stigma positioned at the level of the throat of the corolla tube are a common feature in homostylous flowers of Rubiaceae and also occurs in *Neohymenopogon* Bennet of the tribe Argostemmateae (Chen and Taylor 2011).

## Supplementary Material

XML Treatment for Leptomischus hiepii
